# The combined value of mpUS and mpMRI-TRUS fusion for the diagnosis of clinically significant prostate cancer

**DOI:** 10.1186/s40644-022-00498-8

**Published:** 2022-10-18

**Authors:** Xin Zhang, Hua Hong, Danyan Liang

**Affiliations:** 1 Department of Ultrasound, Inner Mongolia People’s Hospital, Hohhot, 010017 China; 2 Department of Ultrasound, The Affiliated People’s Hospital of Inner Mongolia Medical University, Hohhot, 010017 China; 3 Department of Medical Imaging and Nuclear Medicine, Inner Mongolia Medical University, Hohhot, 010110 China; 4 Department of Statistics Section, Inner Mongolia People’s Hospital, Hohhot, China

**Keywords:** Prostate cancer, Real-time mpMRI, TRUS fusion biopsy, Contrast-enhanced ultrasonography, Elastography, Multiparametric ultrasound

## Abstract

**Objective:**

To evaluate the combined efficacy of multiparametric ultrasonography (mpUS) and multiparametric magnetic resonance imaging/transrectal ultrasound (mpMRI-TRUS) fusion for detecting clinically significant prostate cancer (csPCa).

**Methods:**

From November 2019 to September 2021, biopsy-naïve patients underwent mpMRI-TRUS fusion imaging combined with mpUS-guided targeted biopsies (TB) and systematic biopsies (SB). To further evaluate the additional diagnostic value of mpUS, the imaging features of 202 focus obtained from fusion imaging were assessed. The diagnostic accuracies of mpMRI-TRUS fusion imaging and the combination of mpMRI-TRUS fusion imaging with mpUS for csPCa were comparatively evaluated.

**Results:**

A total of 202 prostate lesions (160 patients) were included in the final analysis, of which 105 were csPCa, 16 were ciPCa, and 81 were noncancerous. The median patient age was 69 (65–73) years and the median tPSA was 22.07 (11.22–62.80) ng/mL. For csPCa, the detection rate of TB was higher than that of SB (50.0% vs. 45.5%, *p* < 0.05). The imaging characteristics of mpUS in the PCa and non-PCa groups were significantly different (*p* < 0.001). When compared with mpMRI-TRUS fusion imaging, the positive predictive value, false positive rate, and area under the curve (AUC) of csPCa diagnosis by mpMRI-TRUS fusion imaging combined with mpUS increased by 11.30%, decreased by 19.58%, and increased from 0.719 to 0.770 (*p* < 0.05), respectively.

**Conclusion:**

TB can improve the detection rate of csPCa and hence can be effectively used in the diagnosis and risk assessment of csPCa. The mpUS-enriched valuable diagnostic information for mpMRI-TRUS fusion imaging and their combination showed a higher diagnostic value for csPCa, which can guide subsequent clinical treatment.

## Background

The cancer-associated death of prostate cancer (PCa) was ranked the fifth highest among men in 2020 worldwide. In more than half the countries globally, PCa is the most common type of cancer [1]. Several methods are used for detecting PCa, such as determining prostate-specific antigen (PSA) levels, digital rectal examination (DRE), magnetic resonance imaging (MRI), and ultrasound. However, PSA or DRE has a high false positive rate that leads to a large number of unnecessary systematic biopsies (SBs) [2]. MRI offers better anatomical resolution and high accuracy in PCa diagnosis compared with other methods, but MRI guide TB (in-bore TB) is rather time-consuming [3]. In recent years, mpMRI-TRUS fusion imaging has garnered attention, enabling sonographers to perform TB under real-time MRI guidance. Fusion imaging combines the sensitivity and specificity of MRI with the real-time and practicality of TRUS, which can detect PCa more accurately and guide TB. It has been used extensively in several hospitals around the world [4].

Compared with PCa, csPCa is more closely related to mortality, and hence increasing csPCa detection rates is the key to reducing mortality. At the same time, reducing clinically insignificant PCa (ciPCa) detection rates is important for improving patients’ quality of life. Hence, accurately identifying csPCa and providing more effective disease-related information to the clinics is crucial.

New ultrasound technologies such as CEUS and TRES have a higher PCa detection rate than conventional TRUS. These new techniques are immensely helpful in diagnosing and treating PCa [5]. Previous studies have reported that CEUS and TRES could detect PCa more accurately by monitoring vascularization in the prostate tissues as well as the hardness of the tumor [6]. Moreover, they are easily accessible to a urologist for conducting routine PCa examinations. However, only a few studies have reported the accuracy of mpMRI-TRUS fusion imaging. Therefore, for increasing the diagnostic accuracy of csPCa, we aimed to evaluate the combined value of mpUS and mpMRI-TRUS fusion for diagnosing csPCa.

## Materials and methods

### Study design

Between November 2019 and September 2020, biopsy naive men with PSA > 4 ng/mL underwent mpMRI a month before biopsies. All patients provided their signed informed consent. The exclusion criteria included contraindications for MpMRI and mpUS examination and biopsies.

### Fusion of mpMRI and mpMRI-TRUS

mpMRI consists of high-resolution T2-weighted imaging (T2WI), diffusion-weighted MR imaging (DWI), and dynamic contrast-enhanced MRI (DCE-MRI). A GE Signa HDxt 3.0 T superconducting MR scanner and an 8-channel phased-array coil were used. The parameters used in the mpMRI protocol are summarized in Table 1*.* The images were read jointly by 2 radiologists with 10 and 3 years of work experience, respectively. If the results provided by the two scholars were inconsistent, the result submitted by the scholar with greater work experience was used as the final result. The PCa location, size, prostate imaging reporting, Data System Version 2.1 (PI-RADS) score, and PCa invasiveness were described and documented for each patient. Patients with PI-RADS scores of 4 or 5 were considered positive [7]

**Table 1:** Parameters used in the mpMRI protocol

Sequence	T2WI	DWI	DCE
Acquisition plane	axial, coronal and sagittal	Axial	Axial
repetition time(ms)	4,000–5,000	≥ 3000	3.3–5.2
echo time(ms)	100–110	≤ 90	1.6–2.3
Section thickness(mm)	3	3	3
FOV(mm)	200	380	380
b Values (sec/mm2)	-	1500	-
Temporal resolution(s)	-	-	≤ 7

mpMRI Digital Imaging and Communications in Medicine (DICOM)-formatted images were uploaded in the UroNav (Invivo, USA) fusion system in Logiq GE20, which transmits the motion and orientation of the transrectal probe (IC5-9) to the fusion system through a triangulation registration method between the magnetic field generator and the electromagnetic tracking sensor on a probe. The motion of the ultrasound images presented by the probe and MRI images were synchronized to accurately guide the TB.

### CEUS and TRES of the target area

The maximum cross-sectional CEUS and RTE imaging of the target area was performed in real-time and evaluated by the deputy chief physician and above (with more than 8 years of work experience) before the biopsy. CEUS was then performed at a low mechanical index (0.12). SonoVue (2.4 mL; Bracco, Milan, Switzerland) and a 5-mL saline flush were intravenously injected subsequently. Continuous recording was performed for 180 s and then switched to the TRES mode followed by putting adequate pressurize on the prostate. When the square green pressure barcode in the upper left was close to full, it indicated that the technique was exact, after which the above images were saved and the lesions were evaluated.

The following mpUS imaging features of the target area of mpMRI-TRUS fusion imaging were recorded: echo level, edge features, whether the aspect ratio was ≥ 1, whether the capsule had a bulge (the nodule grew close to the capsule, making it bulge, thicken, and irregularly interrupted with the contour asymmetry of the capsule) [8], and whether the blood flow distribution was symmetrical. Enhancement pattern, that is, fast-forward enhancement (peripheral zone nodules compared to the central gland, contralateral half-peripheral zone, or ipsilateral peripheral zone tissue; central gland nodule compared to contralateral central gland tissues, within 10–20 s fast enhance), fast-backward enhancement (rapid washout compared to reference tissue), both (fast-forward followed by a rapid wash-out of the contrast agent)*,* and none. The presence or absence of diffuse enhancement was evaluated (including indistinct demarcation between the peripheral zone and central gland; the fast-forwarding and enhancement intensity of the peripheral zone was nearly identical to that of the central gland; the asymmetric distribution of large blood vessels in the prostate showed low/no enhancement area [9]. Perfusion strength, strain pattern, with or without focal no-strain were assessed. PCa of mpUS was assessed according to the EFSUMB2017 guidelines, WFUMB2017 guidelines, and literature reports [10–14].

### Histopathological analysis

TB was performed using a Bard-MAGNUM (MG1522, C. R. Bard, Covington, GA, USA) automatic biopsy gun and a disposable 18-gauge biopsy needle with a needle length of 20 cm. The target area was defined as follows: 1) for mpMRI or mpUS positive: if the target areas of mpMRI and mpUS were consistent, this area was selected for puncture. If the two target areas were inconsistent, they were punctured separately; 2) if both mpMRI and mpUS were negative, both visible nodules were selected for TB. One or two cores were performed per targeted area and then the fusion system was withdrawn for a sagittal 12-needle SB. Biopsy specimens were individually placed in 10% formalin solution and labeled accordingly. We selected the highest GS score of radical prostatectomy or needle biopsies as the final pathological diagnosis. We defined PCa with GS ≥ 3 + 4 as csPCa and GS = 6 as ciPCa [15].

### Statistical analysis

SPSS 20.0 software was used for statistical analysis. Enumeration data were represented by the number of cases (constituent ratio) and measurement data were represented by means ± standard deviation or median (interquartile range). McNemar Chi-square test was used to compare the csPCa detection rate between TB and SB. The remaining categorical variables were compared by using the Chi-square test. The diagnostic efficacy of mpMRI-TRUS fusion imaging, mpUS, and their combination was assessed by receiver operating characteristic (ROC) curve analysis. The test level α = 0.05 and *p* < 0.05 was considered to indicate statistical significance.

## Results

### General clinical and pathological data

A total of 193 patients agreed to participate in the study, of whom, 33 were not included in the analysis due to puncture failure (13) and imaging failure (20). A total of 160 patients (202 lesions) were finally included in the study, and their clinical data are shown in Table 2.

**Table 2:** General clinical and imaging data of patients

Age^a^	69(65–73)
tPSA(ng/ml)^a^	22.07(11.22–62.80)
PSA density^a^	0.41(0.21–1.24)
Lesion location No.(%)	
CZ	94(46.53%)
PZ	83(41.09%)
CZ + PZ	25(12.38%)
Prostate Volume (ml)	52.12(38.33–72.49)
mean lesion size(mm)	15.2(11.1–24.6)
Number of cores of SB	1908 (87.36%)
Number of cores of TB	276 (12.64%)
TB based on mpMRI only	62(2.84%)
TB based on mpUS only	47(2.15%)
TB based on both	167(7.65%)

*Tpsa* total PSA, *CZ* Transition zone, *PZ* Peripheral zone

^a^Data are described by median (interquartile range)

### Comparison of the diagnostic value of TB and SB

There were 105 csPCa, 16 ciPCa, and 81 non-PCa among the 202 lesions. Their pathological results are shown in Table 3*. *Radical prostatectomy was performed in 20 patients, which included 2 cases with a GS score of 6, 15 cases with a GS score of 7, and 3 cases with a GS score of ≥ 8*.*

**Table 3:** Pathological findings of biopsy and prostatectomy results

Benign No.(%)	81(40.10%)
benign glands	31(15.35%)
Inflammation	26(12.87%)
HGPIN	17(8.42%)
ASAP	4(1.98%)
Atrophy	3(1.49%)
PCa No.(%)	121 (59.90%)
GS 6	16 (7.92%)
GS 7	30 (14.85%)
GS 8–10	75 (37.13%)

*ASAP* atypical small acinar proliferation, *HGPIN* high grade prostatic intraepithelial neoplasia

SB detected 92 csPCa and TB detected 101 csPCa. For the detection rate of csPCa, TB was higher than SB (50.0% vs 45.5%, *p* < 0.05). Among these, TB detected 12 prostate cancers missed by SB, of which 10 were csPCa. SB detected 12 prostate cancers overlooked by TB, of which 9 were ciPCa. TB was upgraded to csPCa in 2 patients diagnosed with ciPCa by SB (Table 4).

**Table 4:** Number of csPCa detected by TB and SB

		TB			*χ*^2^	*P*
		No PCa	ciPCa	csPCa		
	No PCa	82(87.23)	2(28.57)	10(9.90)	10.224	0.017
SB	ciPCa	9(12.16)	5(71.43)	2(1.98)		
	csPCa	3(4.05)	0	89(88.12)		
	Total	94	7	101		

Note: There is a statistically significant difference in the csPCa detection of TB and SB (χ^2^value 4.267, *p* = 0.035)

### The mpUS imaging features of mpMRI-TRUS fusion imaging target areas

**Fig. 1:**
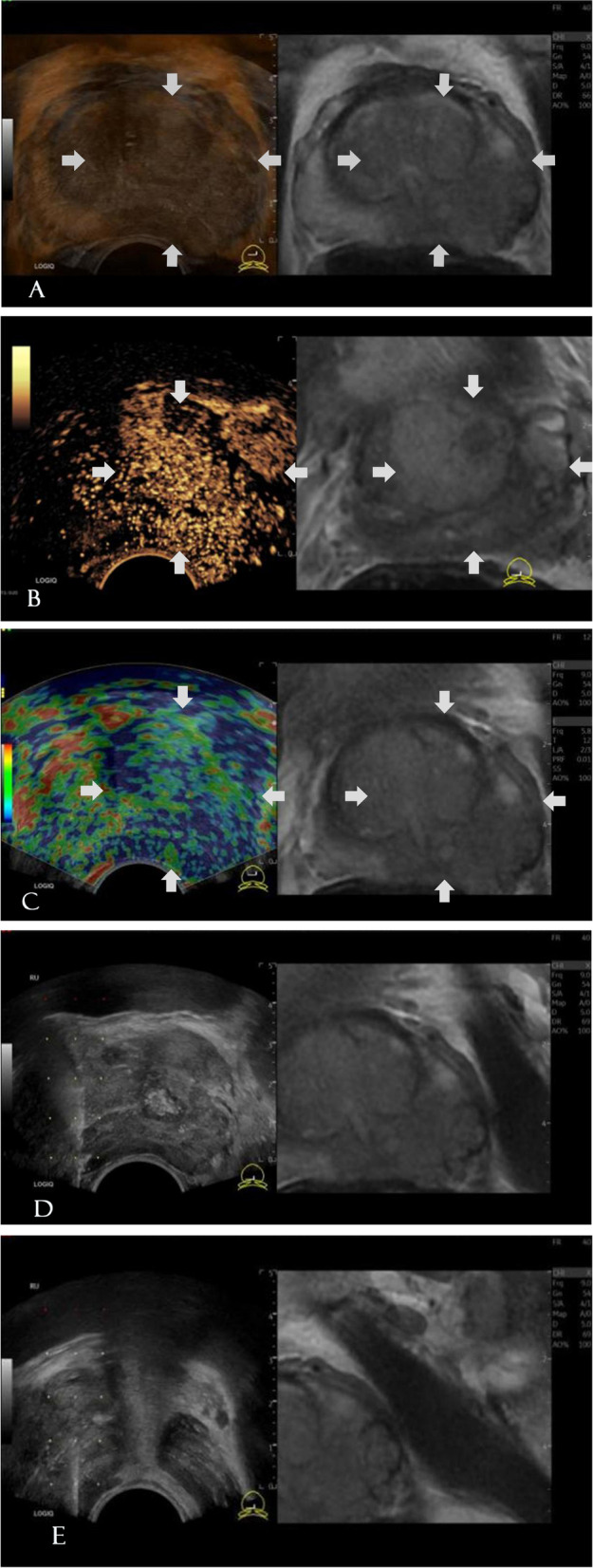
The patient is 69 years old, with TPSA: 560 ng/mL. MpMRI showed suspicious signals in the left central gland and the peripheral zone, mpMRI ( +). Pathological results showing that the lesions were carcinomas, with a Gleason score of 4 + 5 = 9. (A) MpMRI-TRUS fusion, showing the left central and peripheral gland hypoechoic area, with an aspect ratio ≥ 1 and the sign of capsule bulge. (B) CEUS in the targeted area showing fast-forward enhancement (18 s) and hyperperfusion. (C) TRES of the targeted area showing asymmetric, focal non-strain (blue areas entirely covering the entire lesion and beyond the lesion). (D, E) Punctures of the targeted area

**Fig. 2:**
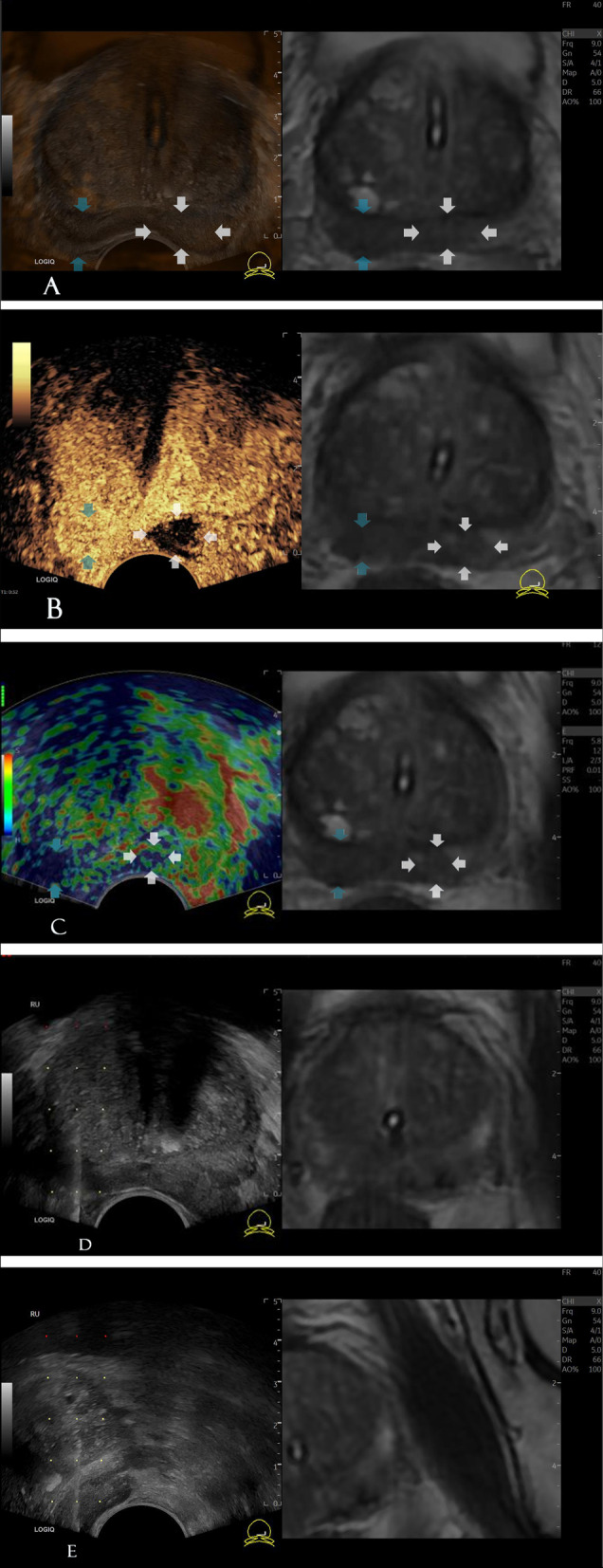
The patient is 78 years old, with TPSA: 62.43 ng/mL. MpMRI showing suspicious signals in the 6–8 O'clock direction of the right peripheral zone (blue arrow), mpMRI ( +). MpMRI showing a nodule in the 5 O'clock direction of the left peripheral zone (white arrow), mpMRI (-). Pathological results implying that the lesions in the right and left peripheral zones are carcinomas, with a Gleason score of 3 + 4 = 7 and 4 + 3 = 7, respectively. (A) MpMRI-TRUS fusion showing the bilateral peripheral gland hypoechoic area, with the sign of capsule bulge in the left peripheral zone. (B) CEUS in the targeted area showing fast-forward and backward enhancements (13 s) as well as hyperperfusion in the right peripheral zone. CEUS in the targeted area showing nonperfusion in the left peripheral zone. (C) TRES of the targeted area showing asymmetric, focal non-strain (the area in the middle of the lesion is blue and the surrounding tissue is green). (D, E) Punctures of the targeted area

Of the 202 lesions, 121 displayed PCa mpUS imaging features:

- 1) B-mode: hypoechoic (61.16%), isoechoic (33.06%), irregular edges (38.02%), aspect ratio ≥ 1 (61.98%), signs of capsule bulge (71.07%), and an asymmetric blood flow distribution (56.20%).
- 2) CEUS: fast-forward enhancement (23.14%) or fast forward and backward enhancement(41.32%). It showed hyperperfusion (45.45%), hypoperfusion/nonperfusion (18.18%), and diffuse enhancement (66.12%).
- 3) TRES: measured as focal non-strain or asymmetric strain (72.73%) and measured as symmetric heterogeneous strain (17.36%) (Table 5, Figs. 1, 2).

**Table 5:** The mpUS imaging features of mpMRI-TRUS fusion imaging target areas

Imaging features	grouping	Number of patients(n)	Pathology results		*Χ*^*2*^	*P*
			benign(%)	PCa(%)		
echo level	hypoechoic	89	15 (18.52)	74 (61.16)	36.260	** < 0.001**
	isoechoic	99	59 (72.84)	40 (33.06)		
	hypoechoic	14	7 (8.64)	7 (5.79)		
edge features	regular edges	148	73 (90.12)	75 (61.98)	19.616	** < 0.001**
	irregular edges	54	8 (9.88)	46 (38.02)		
aspect ratio	≥ 1	110	35 (43.21)	75 (61.98)	6.895	**0.009**
	< 1	92	46 (56.79)	46 (38.02)		
capsule bulge	positive	107	21 (25.93)	86 (71.07)	39.701	** < 0.001**
	negative	95	60 (74.07)	35 (28.93)		
blood flow distribution	symmetrical	115	62 (76.54)	53 (43.80)	21.213	** < 0.001**
	asymmetric	87	19 (23.46)	68 (56.20)		
enhancement pattern	fast-forward enhancement	32	4 (4.94)	28 (23.14)	-	** < 0.001**
	fast-backward enhancement	4	3 (3.70)	1 (0.83)		
	none	93	51 (62.96)	42 (34.71)		
	both	73	23 (28.40)	50 (41.32)		
perfusion strength	hypoperfusion/nonperfusion	26	4 (4.94)	22 (18.18)	22.056	** < 0.001**
	normal perfusion	100	56 (69.14)	44 (36.36)		
	hyperperfusion	76	21 (25.93)	55 (45.45)		
diffuse enhancement	positive	99	19 (23.47)	80 (66.12)	35.332	** < 0.001**
	negative	103	62 (76.54)	41 (33.88)		
strain pattern	homogeneous strain	20	8 (9.88)	12 (9.92)	12.788	**0.002**
	symmetric heterogeneous strain	53	32 (39.51)	21 (17.36)		
	asymmetric strain	129	41 (50.62)	88 (72.73)		
focal no-strain	positive	129	41 (50.62)	88 (72.73)	10.277	**0.001**
	negative	73	40 (49.38)	33 (27.27)

The 81 non-PCa mpUS imaging features showed the following:

- 1) B-mode: isoechoic (72.84%), hypoechoic (18.52%), regular edge (90.12%), aspect ratio < 1 (56.79%), no signs of capsule bulge (74.07%), and an symmetrical blood flow distribution (76.54%).
- 2) CEUS: no fast forward or backward enhancement (62.96%), fast forward and backward (28.40%), normal perfusion (69.14%), and hyperperfusion (25.93%).
- 3) TRES: homogeneous or symmetric heterogeneous strain (49.39%) as well as asymmetric strain (50.62%) (Table 5).

The proportions of the abovementioned characteristics in PCa patients were higher than those in the non-PCa subjects (*p* < 0.001).

### mpMRI-TRUS fusion imaging combined with mpUS to diagnose csPCa

Of the 202 lesions, 121 were diagnosed positive by mpMRI-TRUS fusion imaging alone, of which 85 were csPCa, 7 were ciPCa, and 29 were noncancerous. There were 123 positive lesions diagnosed by mpUS, of which 88 were csPCa, 4 were ciPCa, and 31 were noncancerous. There were 92 positive lesions diagnosed by mpMRI-TRUS fusion imaging combined with mpUS, of which 75 were csPCa, 4 were ciPCa, and 13 were noncancerous.

**Fig. 3:**
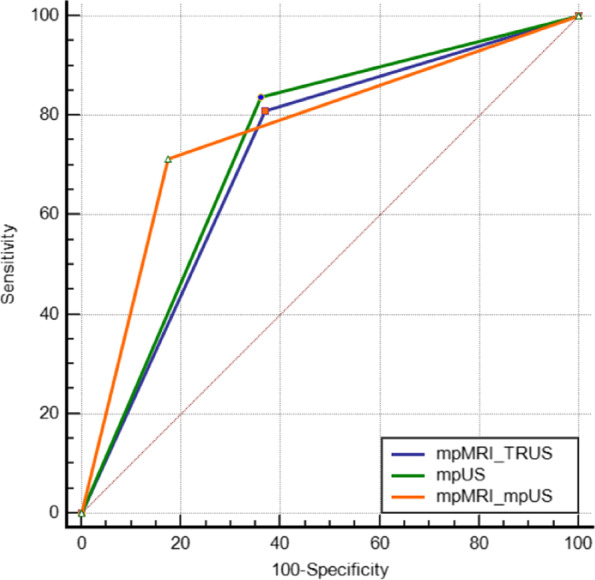
The ROC curves of different imaging modalities in the diagnosis of csPCa

When mpMRI-TRUS fusion imaging was used alone to diagnose csPCa, the sensitivity was 80.95%, specificity was 62.89%, positive predictive value was 70.20%, negative predictive value was 75.30%, and AUC was 0.719 (95% CI: 0.652, 0.780). When mpMRI-TRUS fusion imaging was used in combination with mpUS to diagnose csPCa, the sensitivity was 71.43%, specificity was 82.47%, positive predictive value was 81.50%, negative predictive value was 72.70%, and AUC was 0.770 (95% CI: 0.705, 0.826). Please refer to Table 6 and Fig. 3 for more details.

**Table 6:** Comparison of the efficacy of different imaging modalities in the diagnosis of csPCa

	AUC(95% *CI*)	SE	Sen	Spe	YI	PPV	NPV	Z	P
mpMRI-TRUS	0.719 (0.652 ~ 0.780)	0.0313	80.95%	62.89%	43.84%	70.20%	75.30%	7.007	*p* < *0.001*
mpUS	0.739 (0.672 ~ 0.798)	0.0304	83.81%	63.92%	47.73%	71.50%	78.50%	7.839	*p* < *0.001*
mpMRI-mpUS^*^	0.770 (0.705 ~ 0.826)	0.0294	71.43%	82.47%	53.90%	81.50%	72.70%	9.153	*p* < *0.001*

^*^indicates a *p* value < 0.05 compared to mpMRI-TRUS

## Discussion

According to the 2020 guidelines of the European Association of Urology, mpMRI should be used as a routine application before prostate biopsy [16]. Meanwhile, mpMRI-TRUS fusion imaging has been advocated for clinical application. Xie [17] analyzed the literature on mpMRI-TRUS fusion imaging from 2012 to 2021 and showed that the fusion imaging-guided TB had a higher detection rate of csPCa. Similarly, we confirmed that TB was higher than SB for detecting csPCa (p < 0.05) but our results included the TB guided by mpUS. TB detected 12 PCa missed by SB, 10 of which were csPCa. If only TB was used, 12 (9.9%) PCa would be missed, including 9 ciPCa and only 3 csPCa (2.5%). TB upgraded two patients diagnosed with ciPCa in SB to csPCa. Therefore, TB could detect csPCa more effectively than SB.

Over-diagnosis of PCa is a common problem in the medical profession. One of the reasons is that SB based on abnormal PSA and DRE has the disadvantage of over-detecting ciPCa, missing the diagnosis of csPCa, and underestimating the invasion of cancer foci [18]. To avoid over-diagnosis and treatment of PCa, increase the long-term quality of life of patients, and improve the accuracy of biopsy, it is imperative to diagnose csPCa accurately. We found that TB improves the detection rate of csPCa, which is valuable for clinical diagnosis and risk assessment of csPCa so that patients who benefit from treatment more than observation and follow-up can be effectively selected. Thus, TB has a considerable guiding significance for clinical treatment.

The Cochrane database of systematic reviews suggests that the MRI pathway is superior to SB in making a precise diagnosis of csPCa, albeit it still misses some csPCa. Therefore, it is imperative to conduct further research to resolve this concern [19]. To improve the detection of prostate cancer lesions, we performed CEUS and TRES imaging feature analysis based on the target region of mpMRI-TRUS fusion imaging. Our results suggested that the mpUS characteristics between the PCa and non-PCa groups were statistically significant (*p < *0.001), which is similar to the observations reported by Shinohara, Sano, et al. [20, 21]. With regard to two-dimensional ultrasonography, the literature has well documented that 80% of PCa emerges in the peripheral zone of the prostate and frequently has irregular edges compared with benign prostatic hyperplasia (BPH) due to aggressive growth [22]. The inward spread of PCa was opposed by the surgical capsule between the central gland and the peripheral zone. As a result, they tended to spread outward. The capsular protrusion, thickening, irregular interruptions, and contour asymmetry were associated with capsular invasion. When PCa continues to grow, it breaks through the surgical capsule and grows inward and upward [23], which may be why the aspect ratio was ≥ 1. BPH showed different echoes according to the different components of glands and stroma, most of which are isoechoic with regular edges. Big nodules in the peripheral zone tend to show the characteristics of a capsular bulge, but, in the central glands, they depended on the location, which usually contains normal tissues between them and the capsule. Microvascularization and arteriovenous shunts are a part of tumor-specific pathophysiological processes characterized by high microvessel density that is highly tortuous, disordered, and irregular with shunts. Therefore, PCa is characterized by early contrast agent enhancement and chaotic vascular structure caused by excessive arterial formation, which is manifested as a fast forward enhancement, fast backward enhancement, hyperperfusion, and diffuse enhancement on CEUS. The reduced cross-sectional area of the functional blood vessels in the tumor tissues increased the tortuosity and flow resistance and diminished the perfusion, which also manifested as hypoperfusion/nonperfusion [24]. When compared with PCa, BPH mainly occurs in the central gland, and its enhancement pattern was similar to that of the prostate tissues, which enhanced and disappeared synchronously with the prostate tissue. Some researchers have studied surgical specimens and found fresh blood vessel regeneration in BPH nodules. The blood supply of the external glands is relatively reduced because of the compression of the enlarged internal glands. Therefore, BPH can also manifest as fast forward and backward enhancement as well as hyperperfusion. In the benign tissues, capillaries are mostly confined to the peri-glandular stroma close to epithelial cells, rather than being irregularly distributed as in tumor foci. Increased extracellular matrix deposition and greater adherence to the surrounding tissues contribute to an increase in the hardness of the cancerous tissues when compared to that in the normal tissues; as a result, it manifests as an area with less strain or even no strain on TRES [14]. BPH tissue is less stiff, hence the strain is also relatively homogeneous and symmetrical.

In this study, we performed TB of 31 lesions that were reported negative by MRI but positive by mpUS imaging features, with pathological findings of 13 csPCa and 18 non-PCa. The additional use of mpUS in the targeted region increased the detection of csPCa by 12.4% (13/105). The additional use of mpUS examinations in the targeted area improved the detection of csPCa and provided more efficient diagnostic information for fusion imaging.

To the best of our knowledge, there are only a few studies on the combined application of MpMRI-TRUS fusion imaging and mpUS. Maxeiner et al. investigate whether mpUS can further characterize mpMRI-suspected lesions through fusion and found that mpUS are strong predictors of PCa detection and PI-RADS 5 prediction; therefore, mpUS can be used as an additional tool as well as to reassure the PI-RADS score [25]. However, instead of assessing a combination, the researchers assessed the value of each method individually for diagnosing PCa. Brock et al. found that the fusion of mpMRI with TRES could improve the visualization of PCa lesions when compared to that by MRI alone [26]. Pepe evaluated 21 patients with negative DRE and a past negative biopsy, but with elevated PSA values and concluded that mpUS did not improve the accuracy of TB in diagnosing csPCa, which is inconsistent with our results [27]. First, the inclusion criteria are different from ours, which results in the lack of evaluation of some cases, especially those on the biopsy of naive patients. Second, they did not perform TB in the CEUS or TRES positive areas, therefore it is unknown whether mpUS would have been diagnosed csPCa that were missed by TB. Third, the false negative rate of mpMRI for csPCa (4/21 cases) has not been correlated to the mpUS findings. Finally, the sample sizes are too small, requiring the evaluation of a greater number of cases to reach a concrete conclusion.

Our results showed that, when compared with mpMRI-TRUS fusion imaging alone, the combined diagnosis of csPCa increased the positive predictive value by 11.30% and reduced the false positive rate by 19.58%. When mpMRI-TRUS fusion imaging was used alone, the false positive rate was 37.11% (36/97). The additional use of mpUS in mpMRI-TRUS fusion imaging can reduce the false positive rate of diagnosing csPCa to 17.53% (17/97), indicating that if mpMRI-TRUS is negative and mpUS does not show suspicious signs, the probability of excluding non-csPCa is 82.47%. Therefore, when compared with mpMRI-TRUS fusion imaging alone, the combined method can more effectively diagnose csPCa (AUC 0.719–0.770, *p* < 0.05).

A single imaging method demonstrates limited value in diagnosing cancers. It needs to be combined to maximize the diagnosis of csPCa, which can give patients the most accurate disease information and further guide the clinical treatment. mpUS can be expected to detect plausible characteristics of PCa and provide additional information for patients that can be helpful for their treatment. In the future, further research is required to determine the role of fusion imaging in these patients and imaging at the molecular imaging level to determine which patients may benefit the most from this diagnostic procedure.

The disadvantages of this study are as follows: First, only 20 patients underwent radical prostatectomy and for the remaining 140 patients, we used puncture results as the gold standard. All patients involved underwent biopsies for the first time. Therefore, if the patient’s biopsy result was negative, radical prostatectomy was not performed, leading to the missed diagnosis of low-grade and some advanced PCa to some extent. However, radical prostatectomy is not always prescribed for every patient owing to more complications and elaborate surgical criteria. Second, the high dependence of the PI-RADS distribution on the prevalence of csPCa is the reason for the lack of consensus on PI-RADS category 3 or 4 as positive. In European biopsy-naïve patients, the percentage of PI-RADS 3 potentially indicates the “certainty” of diagnosis and thus that of image quality and reading. However, according to our results, compared with PI-RADS category 3, PI-RADS category 4 produced a better result. Another study reported that the best cutoff for differentiating benign lesions from PCa was PI-RADS category 3. The best cutoff for differentiating low-risk PCa from csPCa was PI-RADS category 4. Therefore, for csPCa evaluation, we used PI-RADS category 4 or more as positive [28].

However, unlike some previous studies, we performed pre-biopsy MRI for patients without contraindications to avoid selection bias instead of biopsy based on MRI risk assessment. Moreover, we not only examined mpMRI-TRUS fusion imaging but also combined it with mpUS. As a result, the imaging features of the fusion region were further characterized, which was deemed valuable for the accurate localization of PCa and the subsequent puncture process. By combining different techniques, the clinical detection limitations of each imaging modality can be balanced. In the future, we believe that this imaging modality can become a valuable tool for the clinical diagnosis of prostate cancer and further guide clinical decision-making.

## Conclusion

We found that TB can be used to improve the detection rate of csPCa, and thus, effectively used in the diagnosis and risk assessment of csPCa. The mpUS characteristics of the target areas can provide valuable diagnostic information for mpMRI-TRUS fusion imaging, which can be helpful for the further visualization of PCa foci. Eventually, we believe that the combination of mpUS with mpMRI-TRUS fusion imaging possesses a higher diagnostic value for csPCa and can provide valuable guidance for subsequent clinical treatment.

## Data Availability

All data generated or analyzed during this study are included in this published article.

## Abbreviations

MpMRI-TRUS: Multiparametric magnetic resonance imaging/transrectal ultrasound
MpUS: Multiparametric ultrasonography
PSA: Prostate specific antigen
DRE: Digital rectal examination
PCa: Prostate cancer
csPCa: Clinically significant prostate cancer
ciPCa: Clinically insignificant prostate cancer
TB: Targeted biopsies
SB: Systematic biopsies
GS: Gleason score
DWI: Diffusion weighted MR imaging
DCE-MRI: Dynamic contrast enhanced MRI
PI-RADS: Prostate imaging-reporting and data system
ADC: Apparent diffusion coefficient
CEUS: Contrast-enhanced ultrasonography
TRES: Transrectal elastosonography
ROI: Region of interest
MI: Mechanical index

## References

[1] Siegel RL, Miller KD, Fuchs HE, Jemal A (2022). Cancer statistics, 2022. CA Cancer J Clin.

[2] Borghesi  M, Bianchi  L, Barbaresi  U, Vagnoni  V, Corcioni  B , Gaudiano  C (2021). Diagnostic performance of MRI/TRUS fusion-guided biopsies vs. systematic prostate biopsies in biopsy-naïve, previous negative biopsy patients and men undergoing active surveillance. Minerva Urol Nephrol.

[3] Del Monte M, Cipollari S, Del Giudice F, Pecoraro M, Bicchetti M, Messina E (2022). MRI-directed biopsy for primary detection of prostate cancer in a population of 223 men: MRI In-Bore vs MRI-transrectal ultrasound fusion-targeted techniques. Br J Radiol.

[4] Derigs F, Doryumu S, Tollens F, Nörenberg D, Neuberger M, von Hardenberg J (2022). A prospective study on inter-operator variability in semi-robotic software-based MRI/TRUS-fusion targeted prostate biopsies. World J Urol.

[5] Zhao YX, Yao GL, Sun J, Wang XL, Wang Y, Cai QQ (2021). Nomogram incorporating contrast-enhanced ultrasonography predicting time to the development of castration-resistant prostate cancer. Clin Med Insights Oncol.

[6] Tyloch DJ, Tyloch JF, Adamowicz J, Juszczak K, Ostrowski A, Warsiński P (2018). Elastography in prostate gland imaging and prostate cancer detection. Med Ultrason.

[7] Turkbey  B, Rosenkrantz AB, Haider  MA, Padhani  AR, Villeirs  G, Macura  KJ (2019). Prostate imaging reporting and data system version 2.1: 2019 update of prostate imaging reporting and data system version 2. Eur Urol.

[8] Smith JA (1996). Transrectal ultrasonography for the detection and staging of carcinoma of the prostate. J Clin Ultrasound.

[9] Jung EM, Weber MA, Wiesinger I (2021). Contrast-enhanced ultrasound perfusion imaging of organs. Radiologe.

[10] Mannaerts CK, Engelbrecht MRW, Postema AW, van Kollenburg RAA, Hoeks CMA, Savci-Heijink CD (2020). Detection of clinically significant prostate cancer in biopsy-naïve men: direct comparison of systematic biopsy, multiparametric MRI- and contrast-ultrasound-dispersion imaging-targeted biopsy. BJU Int.

[11] Postema AW, Gayet MCW, van Sloun RJG, Wildeboer RR, Mannaerts CK, Savci-Heijink CD (2020). Contrast-enhanced ultrasound with dispersion analysis for the localization of prostate cancer: correlation with radical prostatectomy specimens. World J Urol.

[12] Sidhu PS, Cantisani V, Dietrich CF, Gilja OH, Saftoiu A, Bartels E (2018). The EFSUMB guidelines and recommendations for the clinical practice of contrast-enhanced ultrasound (CEUS) in non-hepatic applications: update 2017 (Short Version). Ultraschall Med.

[13] Barr RG, Cosgrove D, Brock M, Cantisani V, Correas JM, Postema AW (2017). WFUMB guidelines and recommendations on the clinical use of ultrasound elastography: part 5. Prostate Ultrasound Med Biol.

[14] Kamoi K, Okihara K, Ochiai A, Ukimura O, Mizutani Y, Kawauchi A (2008). The utility of transrectal real-time elastography in the diagnosis of prostate cancer. Ultrasound Med Biol.

[15] Kasivisvanathan V, Rannikko AS, Borghi M, Panebianco V, Mynderse LA, Vaarala MH (2018). MRI-targeted or standard biopsy for prostate-cancer diagnosis. N Engl J Med.

[16] Mottet N, van den Bergh RCN, Briers E, Van den Broeck T, Cumberbatch MG, De Santis M, et al. EAU-EANM-ESTRO-ESUR-SIOG Guidelines on Prostate Cancer-2020 Update. Part 1: Screening, Diagnosis, and Local Treatment with Curative Intent. Eur Urol. 2021;79(2):243–262. 10.1016/j.eururo.2020.09.042 33172724

[17] Xie J, Jin C, Liu M, Sun K, Jin Z, Ding Z (2022). MRI/Transrectal ultrasound fusion-guided targeted biopsy and transrectal ultrasound-guided systematic biopsy for diagnosis of prostate cancer: a systematic review and meta-analysis. Front Oncol.

[18] Moe A, Hayne D (2020). Transrectal ultrasound biopsy of the prostate: does it still have a role in prostate cancer diagnosis?. Transl Androl Urol.

[19] Drost FH, Osses DF, Nieboer D, Steyerberg EW, Bangma CH, Roobol MJ (2019). Prostate MRI, with or without MRI-targeted biopsy, and systematic biopsy for detecting prostate cancer. Cochrane Database Syst Rev.

[20] Shinohara K, Wheeler TM, Scardino PT (1989). The appearance of prostate cancer on transrectal ultrasonography: correlation of imaging and pathological examinations. J Urol.

[21] Sano F, Terao H, Kawahara T, Miyoshi Y, Sasaki T, Noguchi K (2011). Contrast-enhanced ultrasonography of the prostate: various imaging findings that indicate prostate cancer. BJU Int.

[22] Mitterberger M, Horninger W, Aigner F, Pinggera GM, Steppan I, Rehder P (2010). Ultrasound of the prostate. Cancer Imaging.

[23] Hernandez AD, Smith JA (1990). Transrectal ultrasonography for the early detection and staging of prostate cancer. Urol Clin North Am.

[24] Huang H, Zhu ZQ, Zhou ZG, Chen LS, Zhao M, Zhang Y (2016). Contrast-enhanced transrectal ultrasound for prediction of prostate cancer aggressiveness: The role of normal peripheral zone time-intensity curves. Sci Rep.

[25] Maxeiner A, Stephan C, Durmus T, Slowinski T, Cash H, Fischer T (2015). Added value of multiparametric ultrasonography in magnetic resonance imaging and ultrasonography fusion-guided biopsy of the prostate in patients with suspicion for prostate cancer. Urology.

[26] Brock M, Roghmann F, Sonntag C, Sommerer F, Tian Z, Löppenberg B (2015). Fusion of magnetic resonance imaging and real-time elastography to visualize prostate cancer: a prospective analysis using whole mount sections after radical prostatectomy. Ultraschall Med.

[27] Pepe P, Pepe L, Panella P, Pennisi M. Can multiparametric ultrasound improve cognitive MRI/TRUS fusion prostate biopsy. Arch Ital Urol Androl. 2020;92(2). 10.4081/aiua.2020.2.89 32597106

[28] Woo S, Suh CH, Kim SY, Cho JY, Kim SH (2017). Diagnostic performance of prostate imaging reporting and data system version 2 for detection of prostate cancer: a systematic review and diagnostic meta-analysis. Eur Urol.

